# Surgical Management of Pilocytic Astrocytoma of the Optic Nerve: A Case Report and Review of the Literature

**DOI:** 10.1155/2017/4283570

**Published:** 2017-12-03

**Authors:** Ifeoluwa Apanisile, Tamás Karosi

**Affiliations:** Department of Otolaryngology and Head and Neck Surgery, B-A-Z County Central Hospital and University Teaching Hospital, Miskolc, Hungary

## Abstract

Optic nerve astrocytomas (ONAs) are frequent types of optic nerve gliomas (ONGs), which can affect the visual pathway. An 18-year-old male patient was admitted to our department with right-sided intraorbital/retrobulbar swelling, which progressively grew over several months. Clinical examination showed right-sided diplopia, mydriasis, low visual acuity (0.4), exophthalmus (3 cm), epiphora, and severe retrobulbar pain. There was a family history of high-grade (IV) astrocytomas in which two of the family members died due to the disease. Preoperative MRI scan revealed a soft tissue mass around the retrobulbar area of the right eye with intact orbital bony walls. Surgery was performed whereby it was dissected freely from the muscles and was separated from the optic nerve and the globe. Histopathologic analysis confirmed a benign astrocytoma. The follow-up examination revealed no recurrent or residual tumor. A systemic review of the literature indicates that early diagnosis and experienced multidisciplinary management are required in case of unilateral, resectable forms of ONAs with no distant metastasis, in order to provide a long-time survival of patients. Surgical intervention of unilateral ONAs is a relatively safe procedure, allowing complete or partial tumor removal with minimal morbidity and low recurrence rate.

## 1. Introduction

Optic nerve astrocytomas (ONAs) usually affect the visual pathway, mainly in the pediatric age group. In the orbit, ONAs might arise from the optic nerve and tend to be a dura-bound fusiform swelling with distinct imaging and histologic characteristics. These tumors often extend into the optic chiasm and into the frontal brain lobe and may be associated with neurofibromatosis (type I), respectively [1].

Pilocytic astrocytoma (PA) form of ONA is a rare, slow-growing glioma, classified as grade-I tumor by the World Health Organization (WHO) [2] (Table 1). It typically occurs in children and young adults [2]. Only one-third of patients are older than 18 years of age, and only 17% are older than 30 years. There are no clinical features that are unique to these tumors. Signs and symptoms are usually of several months' duration and are directly related to the size, location, and presence of tumor-associated hydrocephalus [2].

The macroscopic appearance of ONAs is typically well-circumscribed, cyst-like mass with a discrete mural nodule. Microscopically, the tumor often reveals a biphasic pattern in which more compact areas composed of bipolar areas and brightly eosinophilic Rosenthal-fibers that alternate with looser, spongier areas with prominent microcysts. Eosinophilic granular bodies are found in both compact and loose areas [2, 3].

The present study reports a case of a young male patient with unilateral ONA in association with no distant metastasis. The neuroradiological and histopathologic findings and multidisciplinary management are discussed in detail.

## 2. Case Report

**Figure 1:**
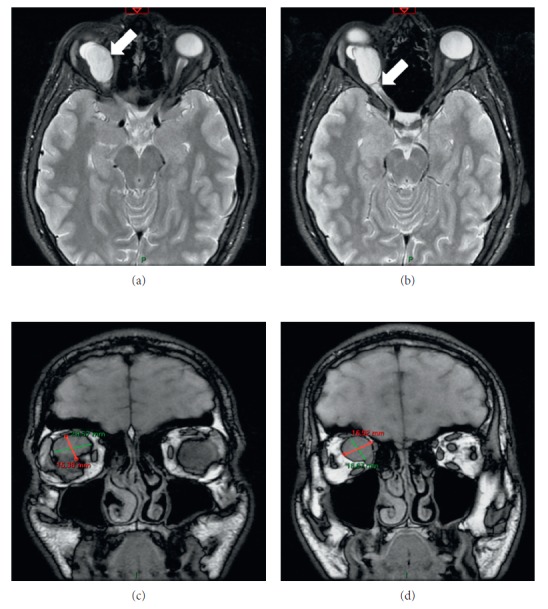
Preoperative MRI scans. (a) A well-defined tumor (white arrow) with vivid signal intensity in the right orbit. (b) The tumor cannot be separated from the optic nerve, followed by the foramen optic (white arrow). (c) The T2-weighted image shows the exact position and size of the tumor. (d) The dorsal edge of the previous shot.

**Figure 2:**
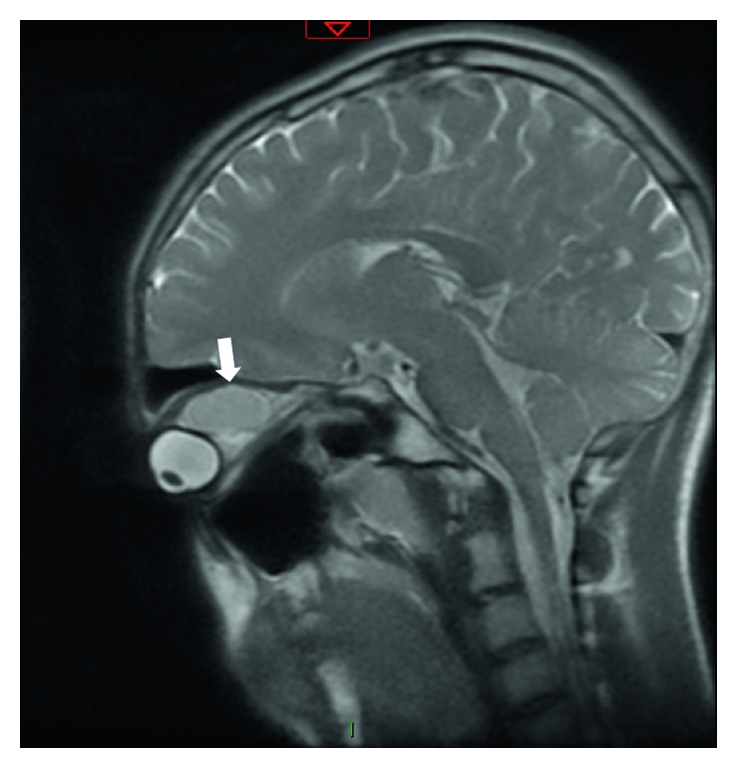
Sagittal T1-weighted MRI. The exact position and extent of the tumor and its relation to the optic nerve are clearly visible. There is a noticeable ocular bulb dislocation (white arrow).

An eighteen-year-old male patient was referred to the outpatient ward of our department on November 2016. He was diagnosed with a right-sided retrobulbar tumor at the ophthalmology department of another medical institution (ophthalmological examination and orbital MRI were performed); however, the ophthalmological team declined to perform the surgical procedure (Figures 1 and 2).

Since our department functions as a tertiary orbital surgery center, after thorough preoperative investigations, we agreed to proceed with the surgery. The symptoms of the patient started about half a year before he was examined at our department. There was no significant information from his past medical history. However, from the family history, it should be noted that his brother died two years earlier due to high-grade (IV) astrocytoma (glioblastoma multiforme), affecting the left side of temporal lobe.

Ophthalmological and clinical examination revealed right-sided diplopia, mydriasis, low visual acuity (0.4), right-sided exophthalmus (3 cm), epiphora, significant eye movement disorders, and severe retrobulbar pain. Exophthalmus was not only evident, but also it clearly dislocates the ocular bulb caudally and laterally. The symptoms started slowly and progressed gradually until eye vision was severely impaired. On the other hand, exophthalmus progressed rapidly.

Preoperative orbital MRI scan revealed the size and exact position of the soft tissue mass around the retrobulbar area of the right eye, its relation to the optic nerve, and the ocular bulb with intact orbital bony walls (Figures 1 and 2).

**Figure 3:**
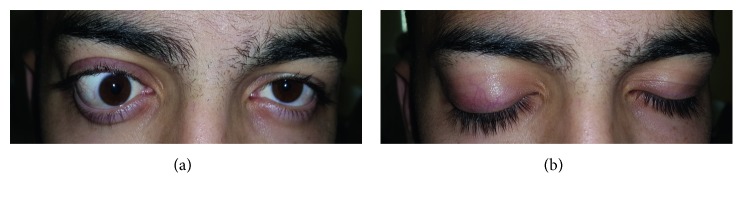
Preoperative images. (a) Observe the right exophthalmus, the downward and outward dislocation of the ocular bulb, which places the pupil's axis toward the medial. Hypoglobus and anisocoria (right-sided mydriasis) are clearly visible symptoms. The movements of the ocular bulb are completely blocked cranially and laterally. (b) The previous images with closed eyes. On the right side, the upper eyelid closure is incomplete.

Figure 3 illustrates the ophthalmological status of opened eyelid and closed eyelid immediately before surgery. After application of preoperative medications, we successfully performed superolateral orbitotomy under general anesthesia. The total duration of the surgery was 69 minutes (see Video files in Supplementary Material available online at https://doi.org/10.1155/2017/4283570). When performing the surgery, we made infraciliary incision to expose the surgical field; thereafter, we bluntly dissected the orbital fat tissue and mobilized the superior rectus muscle, medial rectus muscle, and superior oblique muscle, respectively. Thereafter, we carefully and bluntly separated soft tissues from the muscular structures. Firstly, we applied pressure gauzes, hot saline-soaked gauzes, and sometimes careful and limited usage of electrocoagulation to control bleeding without damaging the surrounding structures. We could observe the tumor infiltrating the dorsal surface of the ocular bulb, closely related to the optic nerve (Supplementary Material).

**Figure 4:**
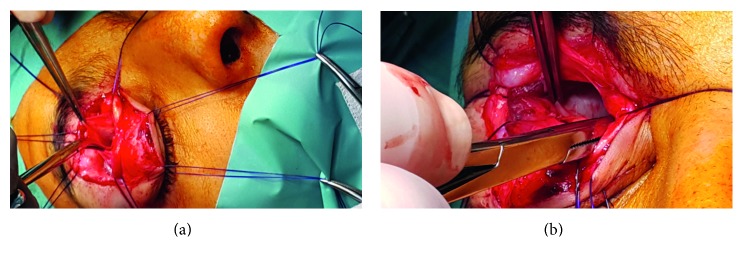
Intraoperative images. (a) We raise the split Tenon-case with clips. The ocular muscles are elevated by several suture threads. (b) Retrobulbar area filled with the bluish-gray color, encapsulated tumor.

**Figure 5:**
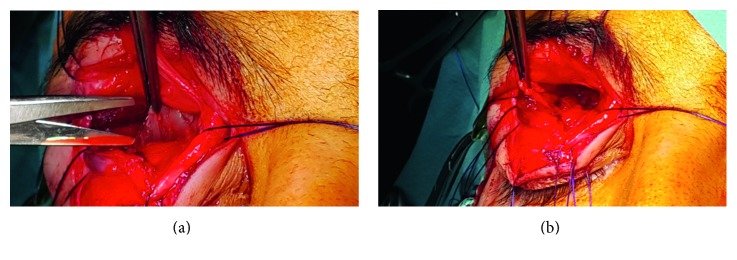
Intraoperative images. (a) By elevating the tumor, we bluntly dissect it carefully from the dorsal surface of the ocular bulb and from the optic nerve. (b) Demonstration of the surgical cavity after successful removal of the tumor.

**Figure 6:**
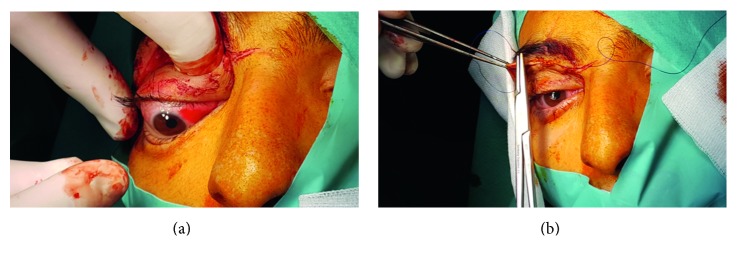
Intraoperative images. (a) Checking ocular bulb movements and relieving any movement constrictions. (b) End stage of the surgery: wound closure with intracutaneous sutures. We could observe the disappearance of the exophthalmus. The mydriasis appeared to increase, and pupil decentralized.

The tumor had a definite encapsulated and cystic character; however, it clearly showed inhomogeneous gelatinous fragments of solid tumor (Figures 4–6). The tumor was carefully removed from the optic nerve, which was followed up to orbital apex. The mobilized tissue was pulled in a ventral direction and then separated from the dorsal surface of the ocular bulb. The surgical cavity was disinfected with polyvidone-iodide solution, and then a tiny rubber drain was placed into the orbit (Supplementary Material).

Prior to wound closure, we checked the eye globe mobility and tension, and we observed that no distension was detectable. The eye muscles were placed in their original anatomical position. The individual soft tissue sections were reconstructed with 4.0 PDS sutures. We reconstructed the orbicularis oculi muscle and the superior levator palpebral muscle with 4.0 Monosyn, using simple sutures. The skin edges were intracutaneously closed with 4.0 Dafilon stitches (Figures 4–6) (Supplementary Material). It should be noted that we did not require ophthalmology consultation during the surgical procedure. Perioperatively, Tobradex eye drops, local ice pack cooling, and systemic antibiotic treatment (Rocephin 2 × 2 g iv.) were used for 3 days.

**Figure 7:**
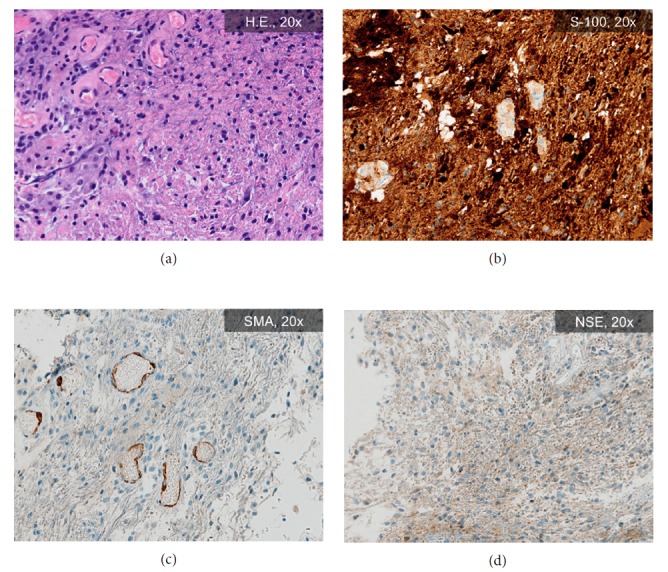
Images of histopathologic examination of the surgical specimen. (a) Hematoxylin-eosin staining represents a solid tumor with several cells showing a vortical pattern. (b) Tumor cells show intense S-100-specific immunoreaction. (c) The smooth muscular actin- (SMA-) specific immunoreaction is practically negative. (d) The neuron-specific enolase- (NSE-) specific immunoreaction represents a strong granular cytoplasmic positivity.

**Figure 8:**
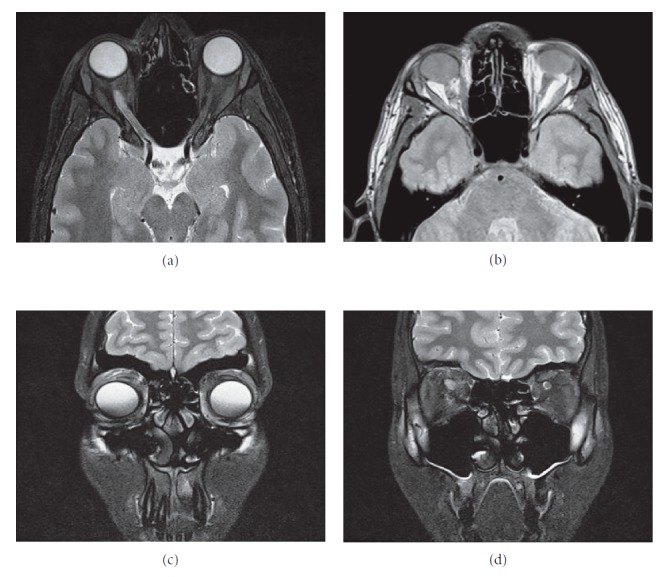
Postoperative MRI scans (3 months). Axial and coronal T1 (a, c) and T2-weighted (b, d) MRI images show normal anatomical conditions with no residual tumor mass.

The eye was covered with photosensitivity pads. During the early postoperative monitoring, chemosis and eyelid swelling were observed; however, it resolved after 1 week of treatment (methyl-prednisolone: 2 × 40 mg iv., and calcium gluconate: 2 × 10 ml iv.). The 8th day after the surgery, the patient was discharged. Histopathologic examination confirmed the diagnosis of benign glial cell tumor based on the expression of neuron-specific enolase (NSE) and synaptophysin (S-100), while cytokeratin AE1/3 reaction and Mib1 markers were negative (Figure 7). Clinical follow-up examination 3 weeks later revealed persistent mydriasis, but exophthalmus, diplopia, and eye movement abnormalities were eliminated. No visual acuity impairment was observed. Postoperative follow-up MRI examination revealed no residual tumor (Figure 8). Informed consent was obtained from the patient for this study and publication of accompanying images and videos. Our study was performed according to the Declaration of Helsinki.

## 3. Discussion

Optic nerve astrocytomas (ONAs) are usually low-grade tumors although their presentation and clinical course are highly variable and unpredictable (Table 1). Gliomas presenting in adulthood are generally very aggressive with rapid decline in vision leading to spread throughout the central nervous system and death [4, 5].

ONGs are extraordinarily rare and seldom seen in routine clinical practice. The pilocytic variant of glioma (astrocytoma) usually affects childhood or early adolescents and is benign and noninvasive in nature [6–8]. These tumors, in most instances, affect the anterior optic pathway and clinically manifest as uniocular visual loss and proptosis [4, 9–11].

There are two possible explanations for the origin of the tumor. One possibility is that brain heterotopia occurred during development, with subsequent sequestration and anatomic discontinuity. This would have been followed by tumor development with effacement of all heterotopic brain tissue. A second possibility is mesenchymal stem cells giving rise to the tumor. The presence of a complete layer of meninges and a cystic cavity would favor a developmental outpouching of embryonic brain rather than a mesenchymal stem cell derivation. It is interesting to note that the glioma was “invading” the surrounding orbital fat through a dural defect. It is worth speculating whether this defect may have represented the point at which the heterotopic tissue was tethered to the rest of the brain before losing its anatomic connection [12]. In general, gliomas of the posterior optic pathways rarely present with proptosis. However, the absence of proptosis does not necessarily preclude sparing of the intraorbital segments of the optic nerve. Optic pathway gliomas are known to be associated with neurofibromatosis type 1; however, our patient did not seem to have this condition [13].

When imaged in cross section, two thirds of all ONAs demonstrate a classic appearance: a cystic mass with an enhancing mural nodule. However, less common appearances are quite nonspecific [14]. A feature specific of this benign neoplasm is that in many cases it displays histologic and imaging features that are commonly seen in higher grade neoplasms and appear incongruous for a slow-growing brain tumor with fairly bland histologic characteristics, such as microvascular proliferation, infiltration of surrounding tissues and structures, intratumoral hemorrhage, intense enhancement on postcontrast images, and leptomeningeal dissemination [15].

Understanding the natural history of these tumors is essential if patients are to receive optimal treatment. For WHO grade I gliomas, the surgical outcome is usually good (Table 1). Poor prognosis is observed for gliomas invading the optic pathways from adjacent parts of the brain, such as the hypothalamus, or suprasellar region [16].

Good quality MRI has almost negated the need for surgical biopsy and is invaluable in the accurate planning of treatment [17]. Surgical excision is only very rarely indicated and is usually limited to those patients with a tumor confined to a single optic nerve and documented radiological evidence of tumor progression toward, but not involving, the optic chiasm. For aggressive tumors, biopsy may be indicated to confirm diagnosis prior to commencing adjuvant therapy [18, 19]. The treatment of ONA is highly individualized, and clinicians should be aware of the benefits and disadvantages of the various treatment options [20].

Although gross total resection of the tumor is often curative, location in critical or deep areas (such as the brainstem and hypothalamus) can render it unresectable and require additional management approaches [21]. Strategies for treating ONA in critical or deep areas, such as chiasmatic and brainstem ONAs, include observation and combinations of surgery, chemotherapy, and radiotherapy. Recent studies have demonstrated that chemotherapy should be the first-line treatment strategy for all patients who have radiological and/or clinical progression, although the best choice of agent(s) remains unclear. Radiotherapy is proven to be effective in combating recurrent and progressive disease, but the side effects of such treatment must always be considered. At present, multifractionated conformal radiotherapy remains the most widely used treatment, but newer techniques of delivering focused radiation continue to advance and may be more beneficial [22]. Although pathological diagnosis remains the gold standard, preoperative diagnosis of tumor behavior and extent is of utmost importance in selecting the appropriate therapeutic management in every single case. Quite often, the diagnosis of ONA is initially made or suggested on the basis of an imaging study. Therefore, it is important for all radiologists to be aware of the many clinical and radiological manifestations of this tumor type.

## 4. Conclusion

Prompt clinical suspicion aided by imaging and histopathologic studies can aid in early diagnosis and subsequent management to improve outcome and reduce morbidity. In this case, surgical excision was done with clinical improvement seen. As mentioned earlier, follow-up MRI examination is planned few months later, and depending on the result, appropriate next management plan will be adequately applied.

## Supplementary Material

Surgical removal of orbital pilocytic astrocytoma via supraciliar approach (parts 1-3).

## Figures and Tables

**Table 1:** Selected CNS gliomas. Comparison of basic histopathologic findings and immunostaining characteristics^*∗*^.

Selected CNS tumor	WHO grade	Histopathology	MIB-1/Ki-67 LI	p53
Pilocytic astrocytoma	I	Biphasic piloid and spongy glial cell regions	<10%	<5%
Diffuse astrocytoma	II	+ Diffuse infiltration and cytoplasmic atypia	<10%	<5%
Anaplastic astrocytoma	III	+ Anaplasia and high mitotic activity	>10%	>5%
Glioblastoma	IV	+ Microvascular proliferation and/or necrosis	>10%	>5%
Ganglioglioma	I	Uniform, monomorphic glial cell and ganglion cell components, diffuse infiltration, and rare mitosises	<5%	<5%
Anaplastic ganglioglioma	III	+ Anaplasia, increased mitotic activity, vascular proliferation, and necrosis	>5%	>5%

^*∗*^Immunostaining characteristics are general guidelines and must be utilised in conjunction with histopathologic features.
